# Pharmacokinetic characterization of fluorocoxib D, a cyclooxygenase-2-targeted optical imaging agent for detection of cancer

**DOI:** 10.1117/1.JBO.25.8.086005

**Published:** 2020-08-28

**Authors:** Maria Cekanova, Sony Pandey, Shelly Olin, Phillip Ryan, Jennifer E. Stokes, Silke Hecht, Tomas Martin-Jimenez, Md. Jashim Uddin, Lawrence J. Marnett

**Affiliations:** a The University of Tennessee, College of Veterinary Medicine, Department of Small Animal Clinical Sciences, Knoxville, Tennessee, United States; b The University of Tennessee, UT-ORNL Graduate School of Genome, Science and Technology, Knoxville, Tennessee, United States; c The University of Tennessee, College of Veterinary Medicine, Department of Biomedical and Diagnostic Sciences, Knoxville, Tennessee, United States; d Vanderbilt University School of Medicine, Vanderbilt Institute of Chemical Biology, Center for Molecular Toxicology and Vanderbilt-Ingram Cancer Center, A. B. Hancock, Jr., Memorial Laboratory for Cancer Research, Departments of Biochemistry, Chemistry and Pharmacology, Nashville, Tennessee, United States

**Keywords:** fluorocoxib, optical imaging, cyclooxygenase-2, pharmacokinetic parameters, dog with naturally occurring cancer, biopsy

## Abstract

**Significance:** Fluorocoxib D, N-[(rhodamin-X-yl)but-4-yl]-2-[1-(4-chlorobenzoyl)-5-methoxy-2-methyl-1H-indol-3-yl]acetamide, is a water-soluble optical imaging agent to detect cyclooxygenase-2 (COX-2)-expressing cancer cells.

**Aim:** We evaluated the pharmacokinetic and safety properties of fluorocoxib D and its ability to detect cancer cells *in vitro* and *in vivo*.

**Approach:** Pharmacokinetic parameters of fluorocoxib D were assessed from plasma collected at designated time points after intravenous administration of 1  mg/kg fluorocoxib D in six research dogs using a high-performance liquid chromatography analysis. Safety of fluorocoxib D was assessed for 3 days after its administration using physical assessment, complete blood count, serum chemistry profile, and complete urinalysis in six research dogs. The ability of fluorocoxib D to detect COX-2-expressing cancer cells was performed using human 5637 cells *in vitro* and during rhinoscopy evaluation of specific fluorocoxib D uptake by canine cancer cells *in vivo*.

**Results:** No evidence of toxicity and no clinically relevant adverse events were noted in dogs. Peak concentration of fluorocoxib D (114.8±50.5  ng/ml) was detected in plasma collected at 0.5 h after its administration. Pretreatment of celecoxib blocked specific uptake of fluorocoxib D in COX-2-expressing human 5637 cancer cells. Fluorocoxib D uptake was detected in histology-confirmed COX-2-expressing head and neck cancer during rhinoscopy in a client-owned dog *in vivo*. Specific tumor-to-normal tissue ratio of detected fluorocoxib D signal was in an average of 3.7±0.9 using Image J analysis.

**Conclusions:** Our results suggest that fluorocoxib D is a safe optical imaging agent used for detection of COX-2-expressing cancers and their margins during image-guided minimally invasive biopsy and surgical procedures.

## 1 Introduction

Optical imaging agents have been synthesized and validated for detection of cancer to guide minimally invasive biopsy or open surgical resections.^1^^–^^8^ These imaging platforms include small molecules, peptides, antibodies, nanoparticles, or affibody labeled with fluorescent dyes.^1^^–^^8^ Optimal targeted contrast agents for clinical application must exhibit safe toxicity profile and have rapid tumor uptake, high tumor-to-background ratio, high specificity and sensitivity, as well as long-term stability.^1^

Cyclooxygenase-2 (COX-2) protein is induced in inflammatory and cancer cells, but not in normal epithelial cells,^9^^–^^20^ which makes COX as an attractive marker in detection of cancer cells.^21^^–^^28^ Derivatives of nonsteroidal anti-inflammatory drugs (NSAIDs) labeled with 5-carboxy-X-rhodamine dyes (fluorocoxibs) ($\lambda_{\mathrm{ex}}=580\;\mathrm{nm}$ and $\lambda_{\mathrm{em}}=605\;\mathrm{nm}$) have been synthesized and evaluated as optical imaging agents for detection of COX-2 in cancer cells.^29^^,^^30^ We and others have proven the selective and specific uptake of fluorocoxib A by COX-2-expressing cancer cells *in vitro* and *in vivo*.^31^^–^^38^ We have also shown that fluorocoxib A has the ability to not only detect the cancer but also has an ability to monitor the tumor’s responses to therapy.^32^ Fluorocoxib D, N-[(rhodamin-X-yl)but-4-yl]-2-[1-(4-chlorobenzoyl)-5-methoxy-2-methyl-1H-indol-3-yl]acetamide, is a decarboxy analog of fluorocoxib A, and a derivative of indomethacin that selectively binds and inhibits the COX-2 enzyme.^39^ It has been shown that fluorocoxib D has improved water solubility as compared to fluorocoxib A^39^ in order to progress clinical translation of this COX-2-targeted optical imaging agent.

Validation of imaging and therapeutic agents mostly relies on the whole-body imaging approach using the rodent cancer models, which is a limiting factor in the effective translation of imaging agents to human clinical applications. Companion animals with naturally occurring cancers are more appropriate animal models used for a validation of imaging agents and therapeutics.^38^^,^^40^^–^^46^ Spontaneous cancers in companion dogs offer a unique model for human cancer biology.^38^^,^^40^^–^^46^ Thus, to further evaluate the promising new optical imaging fluorophore, fluorocoxib D, we investigated its safety and pharmacokinetic properties in healthy research dogs. Further, we validated the selective uptake of fluorocoxib D by COX-2-expressing human cancer cells *in vitro*. We also confirmed specific uptake of fluorocoxib D in histology-confirmed COX-2-expressing cancer during image-guided biopsy procedure using rhinoscopy in client-owned dog *in vivo*.

## 2 Material and Methods

### 2.1 Fluorocoxib D

**Fig. 1:**
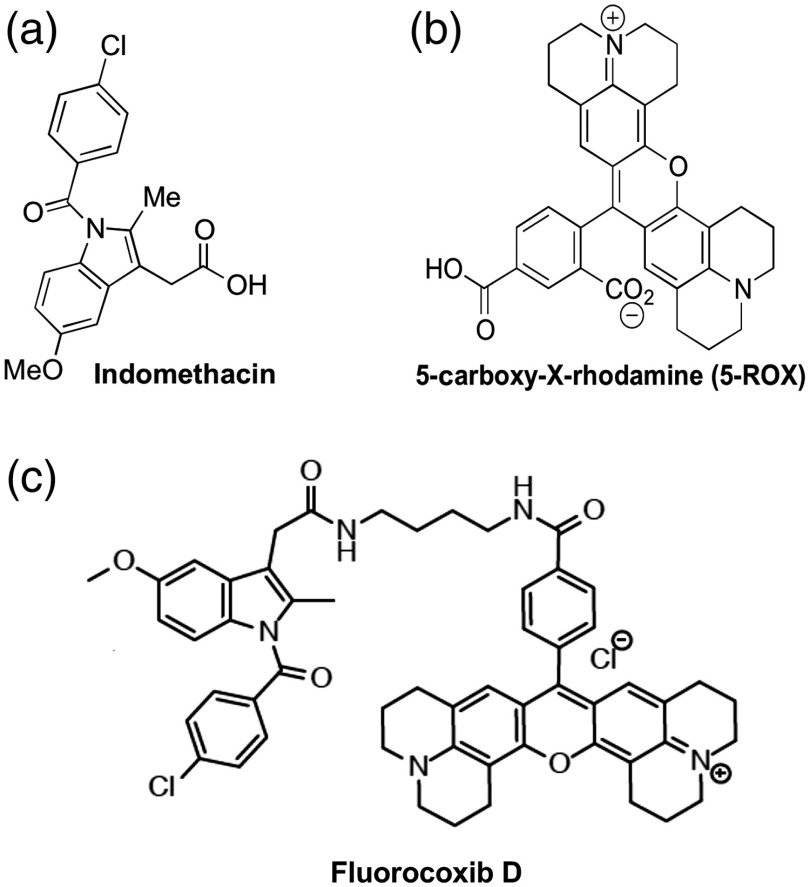
Chemical structure of (a) indomethacin, (b) 5-carboxy-X-rhodamine (5-ROX), and (c) fluorocoxib D.

Fluorocoxib D was synthesized as previously described by Uddin.^39^ The chemical structures of indomethacin, 5-carboxy-rhodamine, and fluorocoxib D along with their potencies as COX-inhibitors are shown in Fig. 1 and Table 1.

**Table 1:** Indomethacin, fluorocoxib A, and fluorocoxib D inhibit COX-1 and COX-2 activities.

	${\mathrm{IC}}_{50}$ (μM)			
	COX1	COX2	Ratio (COX1/COX2)	RAW264.7 macrophages
Indomethacin^47^	0.19±0.13	20.9±10.4	0.009	0.01^29^
Fluorocoxib A^29^	25	0.7	35.7	0.31
Fluorocoxib D^39^	4	0.23	17.4	—

In our *in vivo* experiments, fluorocoxib D was dissolved in dimethyl sulfoxide/ethanol/propylene glycol/saline solution to final concentration of 5  mg/ml under sterile conditions and filtered using a 0.1-μm pore size filter.

### 2.2 Antibodies and Other Reagents

Antibody for COX-2 was obtained from Cayman Chemical Corporation (Ann Arbor, Michigan), and antibody for Ki67 was purchased from Dako (Carpinteria, California). All other chemicals and reagents were purchased from Thermo Fisher Scientific (Pittsburgh, Pennsylvania), unless otherwise specified.

### 2.3 Human Cancer Cell Line

Human 5637 bladder cancer cells were purchased from American Type Culture Collection (ATCC, Manassas, Virginia). Human 5637 cancer cells were maintained in RPMI-1640 media supplemented with 10% fetal bovine serum, 100 I.U. penicillin, and 100  μg/ml streptomycin (complete media) and grown in an atmosphere of 5% ${\mathrm{CO}}_{2}$ at 37°C.

### 2.4 Fluorocoxib D Uptake by Human 5637 Cancer Cells

Human 5637 cells were seeded on four-chamber slides (Nalge Nunc, Rochester, New York) and grown until reached 70% to 80% confluence. Then, the cells were pretreated with or without 10  μM celecoxib for 30 min followed by a treatment with 50 nM fluorocoxib D for an additional 30 min. After this treatment, cells were washed with PBS and incubated for additional 1 h in complete media to remove any unbound fluorocoxib D. Cells were then washed twice with PBS and fixed in 10% formalin for 15 min at room temperature. The nuclei of cells were stained with DAPI for 10 min, and slides were mounted using an aqueous mounting medium. Images of cells were captured by a Leica DMi8 fluorescent microscope with Hamamatsu Orca Flash 4.0LT Digital CMOS Camera (Leica Microsystems Inc., Chicago, Illinois) using Leica Application Suite X software (LASX, Leica).

### 2.5 Research Healthy Dogs

The animal studies were performed in the accordance with the University of Tennessee Institutional Animal Care and Use Committee (UT IACUC)-approved protocols and in an accordance with NIH guidelines. Individually housed research healthy female beagle dogs (Covance Research Product) with weights of 6.7 to 10.3 kg were used to assess the safety and pharmacokinetic parameters of fluorocoxib D administrated a dose of 1  mg/kg over 20 min intravenously (i.v.).

### 2.6 Pilot Safety Study of Fluorocoxib D in Research Dogs

For single dose safety evaluation studies, we administrated fluorocoxib D at 1  mg/kg, i.v. using a preplaced cephalic or saphenous vein catheter over 20 min to six healthy research dogs following protocol as described previously.^38^ Briefly, physical examinations for signs of potential drug toxicity were performed before, during, and daily for 3 days after fluorocoxib D administration. Physical assessment included auscultation of heart and lungs, abdominal palpation, body temperature, appetite, attitude, activity levels monitoring, and other health-related events. Hypersensitivity was evaluated by direct observation of each dog during treatment for clinical signs of an allergic reaction (facial swelling, flushing, urticaria, dyspnea, scratching, and changes of heart rates). Signs of adverse events after fluorocoxib D administration were monitored daily, including vomiting, diarrhea, depression, nausea, and increased salivation. Laboratory evaluations of blood and urine were carried out before and 3 days after fluorocoxib D administration to monitor signs of toxicity. Blood (up to 3 ml) was collected into ethylenediaminetetraacetic acid (EDTA) and heparin-treated tubes, and urine (up to 5 ml) was collected by cystocentesis or from the floor of the runs. The laboratory evaluation consisted of a complete blood count (CBC), serum chemistry profile, and complete urinalysis. The CBC evaluated total white blood cells (WBC) and hematocrit (HCT), in addition to absolute numbers of neutrophils, lymphocytes, monocytes, eosinophils, and platelets. The plasma chemistry profile evaluated blood urea nitrogen (BUN), creatinine, proteins (albumin, globulins, and total proteins), alanine aminotransferase (ALT), total bilirubin, glucose, and electrolytes. The complete urinalysis evaluated urine proteins, glucose, and ketones; a sediment examination and urine-specific gravity were validated as well for any possible signs of renal toxicity of fluorocoxib D. All laboratory tests were carried out at the Veterinary Medical Center of the University of Tennessee in Knoxville.

### 2.7 Pharmacokinetics of Fluorocoxib D in Research Dogs

To determine the pharmacokinetic parameters of fluorocoxib D (1  mg/kg, i.v.) in dogs, blood (2 to 3 ml) was collected from the jugular vein into EDTA-treated tubes at 0, 0.5, 1, 2, 4, 8, and 24 h after compound administration. The blood was centrifuged within 20 min of sample collection. Plasma was removed and stored at −80°C until further HPLC analysis.

### 2.8 HPLC Analysis of Plasma

Levels of fluorocoxib D were determined in collected canine plasma samples using HPLC at Vanderbilt University (JU). The frozen samples were thawed and extracted with acetonitrile. The organic layer was collected, dried, and reconstituted in methanol and water. The unknown samples were quantitated against a 5-point standard curve, which was prepared by mixing known quantities of fluorocoxib D with commercial canine plasma (Sigma-Aldrich) and subjecting the resulting samples to liquid–liquid extraction and HPLC. Samples were analyzed using a reverse-phase column (C18, 5×0.2  cm, Phenomenex) with gradient elution. The mobile phase component A was water, and B was acetonitrile, each containing 0.1% acetic acid. The gradient was 50% B to 90% B over 5 min, followed by a brief hold and return to initial conditions. The flow rate was 0.3  ml/min.

### 2.9 Pharmacokinetic Parameters

The pharmacokinetic parameters for fluorocoxib D were estimated from the plasma concentration-time data by a nonlinear mixed effects approach, as implemented with Monolix 4.1.2 (Lixoft S.A.S., Orsay, France). This approach allowed analyzing data from all animals at the same time and was ideal for relatively sparse datasets like the one used in this study. The plasma clearance (Cl), representing the overall ability of the body to eliminate fluorocoxib D, was determined by scaling its elimination rate (amount per time) by the corresponding plasma concentration level. The volume of distribution (Vd) was defined as the ratio of the total amount of fluorocoxib D in the body to the blood plasma concentration. The Cl and Vd were estimated directly from the study population data. The peak concentration in plasma ($C_{\max}$) and the following pharmacokinetic parameters were obtained by averaging the corresponding post-hoc Bayesian estimates. The individual partial areas under the curve between times 0 and 8 h (${\mathrm{AUC}}_{0-8}$) and the ${\mathrm{AUC}}_{0-\infty }$ with extrapolation to infinity were calculated using the log-linear trapezoidal rule, as implemented with WinNonlin 5.1 (Pharsight, Mountain View, California). The mean residence time (MRT) value was determined as the ratio of the area under the first moment curve over ${\mathrm{AUC}}_{0-\infty }$. The elimination rate constant was determined as the slope obtained by linear regression of the terminal log-linear portion of the concentration versus time curve, and the elimination half-life ($t_{1/2}$) was then calculated. The plasma concentration graphs were generated using calculated averaged plasma concentrations of fluorocoxib D from six dogs after 1  mg/kg i.v. of fluorocoxib D administration performed over 20 min.

### 2.10 Rhinoscopy-Guided Biopsy Sample Collection from a Client-Owned Dog with HNC Mass

A client-owned dog diagnosed with naturally occurring cancer was enrolled in our study through the Veterinary Medical Center by the Internal Medicine Service to validate the selective uptake of fluorocoxib D in cancer cells. The owners signed a consent form for enrolling their dog for our proof-of principal study. A nine-year old female-spayed Golden Retriever dog weighing 35 kg was presented for an acute onset of unilateral right-sided epistaxis of 5-day duration. Previous medical history included a grade 2 soft tissue sarcoma with a high mitotic index that was completely excised from her ventral neck ∼2 years prior with no signs of regrowth. Rhinoscopy and biopsy procedure in the dog was performed by board-certified veterinary internal specialists (SO and JS) in an accordance with a standard veterinary care and the UT IACUC-approved protocol. Fluorocoxib D was administrated i.v. 1  mg/kg over 20 min using a catheter, followed by a half hour uptake before initiation of rhinoscopy procedure. Rhinoscopy was used to obtain biopsy samples guided by uptaken fluorocoxib D in the computed tomography (CT)-identified head and neck cancer (HNC) mass. Rhinoscopy and biopsy procedures in dog were performed under general anesthesia using a 2.7-mm, 30-deg, 18-cm rigid scope (Karl Storz Veterinary Endoscopy) attached to a TRICAM PDD as described previously^38^ using a custom-designed filter system for detection of fluorocoxib D ($\lambda_{\mathrm{em}}$ 645±50  nm). After complete recovery from the rhinoscopic examination and anesthesia in a quiet room, the client-owned dog was returned to its owners. The heat map images with adjusted hue saturation of fluorocoxib D signal (pink color) from normal tissue (upper three images) and HNC tumor (lower three images) obtained during rhinoscopy were generated using Adobe Photoshop CC2019 software (Adobe Acrobat) in order to better visualize the tumor and its margins. Tumor-to-normal tissue ratio (T/N ratio) of fluorocoxib D signal obtained from normal (n=804) and tumor regions of interest (ROI) (n=866) from plot profile images generated from ImageJ software (NIH).

### 2.11 Computed Tomography in a Client-Owned Dog with HNC Mass

CT of head in the client-owned dog was performed using a multislice helical CT scanner (Philips Brilliance-40, Philips International B.V., Amsterdam, Netherlands). A submillimeter multislice dataset of the head was acquired, and images were reconstructed in 0.9- and 4-mm slice thickness utilizing bone and soft tissue algorithms. The acquisition was repeated following intravenous administration of nonionic iodinated contrast medium (Ioversol 350  mg/ml, 2.2  mg/kg [1  mg/lb], Optiray^®^ 350  mg/ml, Tyco Healthcare/Mallinckrodt, Milwaukee, Wisconsin, USA). Images were evaluated by a board-certified veterinary radiologist (SH).

### 2.12 Immunohistochemistry

The biopsy samples obtained during rhinoscopy procedures in dogs were formalin-fixed and paraffin-embedded and sectioned at 7-μm thin sections for histology and immunohistochemistry analysis for detection of COX-2 and Ki67 expression as described previously.^38^ For the antigen retrieval, sodium citrate buffer (10 mM, pH 6.0) was used for unmasking COX-2 and Ki67 proteins. Endogenous peroxidase activity was blocked by 3% hydrogen peroxide, and nonspecific binding of secondary antibody was eliminated by incubation with protein block buffer (Biogenex USA, San Ramon, California) for 30 min at room temperature. Slides were incubated with primary antibodies for COX-2 (1:500) or Ki67 (1:500) overnight at 4°C, followed by incubation with specific secondary antibodies, streptavidin/biotin horseradish peroxidase complex (Biogenex), and visualized by DAB staining. Sections were lightly stained with Mayer’s hematoxylin. The tissue specimens were evaluated using a Leitz DMRB microscope. The images were captured by a DP73 camera attached to microscope using CellSens Standard software (Olympus, Pittsburgh, Pennsylvania).

## 3 Results

### 3.1 Safety of Administration of Fluorocoxib D to Research Dogs

To assess the safety of a single dose of fluorocoxib D, six female healthy research dogs were administered with fluorocoxib D (1  mg/kg, i.v., over 20 min). A physical examination, CBC (Table 2), serum chemistry profile (Table 3), and complete urinalysis (Table 4) confirmed the clinically healthy status of the research dogs at baseline and 3 days after administration of fluorocoxib D. Of note, there were a few parameters that were outside of the normal laboratory reference ranges. Four dogs (dog #1, #3, #4, and #6) had low normal or mild hypoalbuminemia before and after administration of fluorocoxib D; the hypoalbuminemia remained stable in these dogs before and after fluorocoxib D administration (Table 3). One dog (dog #6) developed mild neutropenia 3 days after fluorocoxib D administration but remained afebrile and clinically normal; the cause of neutropenia in this dog was uncertain. The finding of hypoalbuminemia and mild hypocalcemia may reflect a chronic subclinical condition in these dogs. The mild hypernatremia (dog #6, Table 3) likely reflects subclinical dehydration given the well-concentrated urine (urine specific gravity 1.044, Table 4). The trace protein on urine dipstick is likely because the urine is well-concentrated. Three days after fluorocoxib D administration, two dogs (dogs #4 and #6) had bacteriuria (Table 4). Given the absence of pyuria, the bacteriuria could be iatrogenic or represent asymptomatic bacteriuria. The finding of bacteriuria is not thought to be relevant to this acute toxicity study. Adverse events including diarrhea, vomiting, nausea, and loss of appetite were not observed, although one dog exhibited increased salivation during the administration of fluorocoxib D over 20 min, most likely due to irritation from present DMSO as a one of the solvents of fluorocoxib D. Overall, the data indicate that single-dose administration of fluorocoxib D used at the dose of 1  mg/kg was a safe dose for dogs.

**Table 2:** CBC analysis of blood before and 3 days after a single dose of fluorocoxib D (1 mg/kg, i.v.) in six healthy research dogs.

Test analysis		Dog #1	Dog #2	Dog #3	Dog #4	Dog #5	Dog #6	Reference ranges at UTCVM	Units
WBC	Before	6.9	7.4	7	6.8	6.5	6	5.1 to 14	$\times 10^{3}/\mu l$
	After	5.3	5.7	6.6	7.3	7.8	5.3		
HCT	Before	45.1	50.1	44.7	46.4	51.5	45.4	41 to 60	%
	After	45.2	47.2	45.6	47.2	47	48.1		
Abs SEG	Before	4.11	4.42	4.78	4.49	3.72	3.94	2.65 to 9.8	$\times 10^{3}/\mu l$
	After	3.22	2.98	4	4.72	5.07	2.4 L		
Abs BAN	Before	N/A	N/A	N/A	N/A	N/A	N/A	0 to 0.3	$\times 10^{3}/\mu l$
	After	N/A	N/A	N/A	N/A	N/A	0		
Abs LYM	Before	2.15	2.25	1.75	1.76	3.72	1.75	1.1 to 4.6	$\times 10^{3}/\mu l$
	After	1.61	2.05	2.22	1.88	5.07	2.3		
Abs MON	Before	0.28	0.31	0.31	0.38	0.26	0.22	0.165 to 0.85	$\times 10^{3}/\mu l$
	After	0.23	0.3	0.24	0.49	0.38	0.62		
Abs EOS	Before	0.34	0.4	0.15	0.16	0.31	0.08	0 to 0.85	$\times 10^{3}/\mu l$
	After	0.24	0.36	0.14	0.2	0.18	0.17		
PLT EST	Before	N.D.	ADQ	N.D.	N.D.	ADQ	ADQ	ADQ	—
	After	N.D.	N.D.	ADQ	N.D.	N.D.	N.D.		
PLT CNT	Before	N/A	345	N/A	N/A	256	196	147 to 423	$\times 10^{3}/\mu l$
	After	N/A	N/A	241	N/A	N/A	N/A		

Out of reference range values labeled with H-high or L-low in italic.

WBC, white blood cells; HCT, hematocrit; Abs SEG, absolute segmented neutrophils; Abs BAN, absolute band neutrophils; Abs LYM, absolute lymphocytes; Abs MON, absolute monocytes; Abs EOS, absolute eosinophils; PLT EST, platelet estimate; PLT CNT, platelets counts; ADQ, adequate—visual examinations of the blood smears identified an adequate number of platelets; N.D, not determined, samples is clumped and platelet numbers not determine, but ADQ., N/A, not available.

**Table 3:** Chemistry panel of serum before and 3 days after a single dose of fluorocoxib D (i.v., 1 mg/kg) in 6 healthy research dogs.

Test analysis		Dog #1	Dog #2	Dog #3	Dog #4	Dog #5	Dog #6	Reference ranges at UTCVM	Units
BUN	Before	11	15	17	13	15	16	7 to 37	mg/dL
	After	12	17	14	16	17	19		
CREATI	Before	0.7	0.6	0.7	0.6	0.9	0.6	0.3 to 1.1	mg/dL
	After	0.8	0.6	0.8	0.6	0.8	0.6		
TOT. PROT	Before	5.4	5.8	5.8	5.7	5.7	5.2 L	5.4 to 6.8	g/dL
	After	5.4	5.4	5.7	5.6	5.6	5.2 L		
ALBUM	Before	3 L	3.6	3.4	3.3	3.8	3.3	3.2 to 4.3	g/dL
	After	3.1 L	3.4	3.4	3 L	3.5	3.3		
GLOB	Before	2.4	2.2	2.4	2.4	1.9	1.9	1.9 to 3.1	g/dL
	After	2.3	2	2.3	2.6	2.1	2		
GLUCO	Before	101	101	91	87	105	103	82 to 132	mg/dL
	After	94	103	100	91	105	110		
CAL	Before	9.2 L	10.5	9.9 L	9.8 L	10.4	9.3 L	10 to 12	mg/dL
	After	9.5 L	10	9.9 L	9.7 L	10.3	9.4 L		
PHOS	Before	3.3	4.3	3.6	2.6	3.3	2.7	2.5 to 5.9	mg/dL
	After	3.5	4.3	3.3	3.9	3.4	3.6		
ALT	Before	25	24	22	22	22	21	18 to 100	U/L
	After	26	23	26	22	22	25		
SOD	Before	145	146	146	146	148 H	147	141 to 147	mEq/L
	After	144	146	147	146	147	148 H		
POTAS	Before	4.1	4.1	3.9	3.5	3.5	3.3	2.8 to 4.7	mEq/L
	After	3.9	4.2	3.7	3.5	3.8	3.7		
CHLOR	Before	115	111 L	113	110 L	111 L	115	112 to 119	mEq/L
	After	111 L	114	114	113	112	116		
MAG	Before	0/7	0.8	0.8	0.7	0.8	0.7	0.65 to 0.98	mmol/L
	After	0.7	0.8	0.8	0.7	0.7	0.7		
TOT BILI	Before	N/A	0.1	0.1	0.1	0.1	N/A	0.1 to 0.6	mg/dL
	After	0.1	N/A	0.1	N/A	N/A	0.1		

Out of reference range values labeled with H-high or L-low in italic.

BUN, blood urea nitrogen; CREATI, creatinine; TOT PROT, total proteins; ALBUM, albumin; GLOB, globulin; GLUCO, glucose; CAL, calcium; PHOS, phosphorus; ALT, alanine aminotransferase; SOD, sodium; POTAS, potassium; CHLOR, chlorine; MAG, magnesium; TOT BILI, total bilirubin; N/A, not available.

**Table 4:** Analysis of urine samples before and 3 days after a single dose of fluorocoxib D (1 mg/kg, i.v.) in six healthy research dogs.

Test analysis		Dog #1	Dog #2	Dog #3	Dog #4	Dog #5	Dog #6	Reference ranges at UTCVM	Units
SP. GRAV	Before	1.022	1.023	1.05	1.025	1.026	1.027	—	—
	After	1.03	1.039	1.032	1.039	N/A	1.044		
PH	Before	5.5	8.5	7.5	8.5	6.5	7	5 to 9	—
	After	>9 H	6	6	6	N/A	*5.5*		
PRO-TEIN	Before	NEG	NEG	2+	1+	NEG	NEG	NEG-1+	—
	After	2+	NEG	NEG	NEG	N/A	TRACE		
KETO-NES	Before	NEG	TRACE	1+	1+	NEG	1+	NEG	—
	After	NEG	NEG	TRACE	TRACE	N/A	NEG		
GLU-COSE	Before	NEG	NEG	NEG	NEG	NEG	NEG	NEG	mg/dL
	After	NEG	NEG	NEG	NEG	N/A	NEG		
BLD/Hb	Before	NEG	NEG	NEG	NEG	NEG	NEG	NEG	—
	After	NEG	TRACE	TRACE	1+	N/A	NEG		
WBC/HPF	Before	N/A	N/A	N/A	0 to 1	N/A	N/A	0 to 5	#/HPF
	After	0 to 2	0 to 1	0 to 1	0 to 2	N/A	0 to 3		
RBC/HPF	Before	N/A	N/A	N/A	5 to 10	N/A	N/A	0 to 5	#/HPF
	After	0 to 2	*12 to 15*H	*10 to 19*H	5 to 10	N/A	0 to 3		
CASTS	Before	N/A	N/A	N/A	N/A	N/A	N/A	0	#/LPF
	After	N/A	N/A	N/A	N/A	N/A	N/A		

Out of reference range values labeled with H-high or L-low in italic.

SP. GRAV, urine-specific gravity; PH, pH; BLD/Hb, blood/hemoglobin; WBC/HPF, white blood cells per high power field; RBC/HPF, red blood cells per high power field; LPF, low power field; N/A, not available

### 3.2 Pharmacokinetic Characteristics of Fluorocoxib D in Dogs

**Fig. 2:**
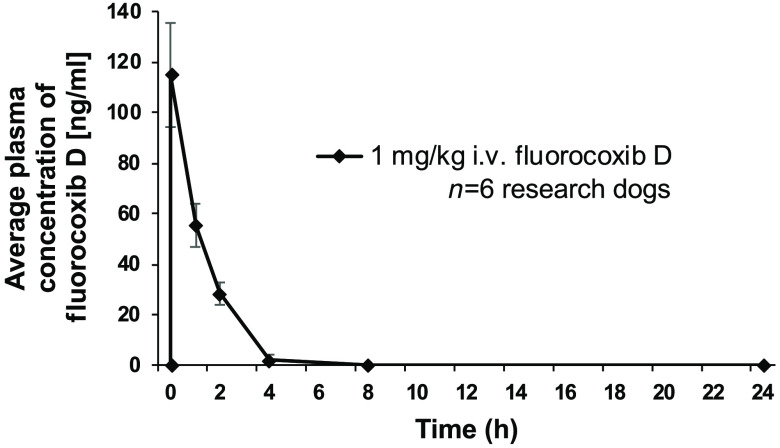
The time course of fluorocoxib D plasma concentrations over 24 h of fluorocoxib D following administration by HPLC analysis. The data represent the mean plasma concentrations of fluorocoxib D (ng/ml) at specific time points following 1  mg/kg i.v. administration over 20 min collected from research dogs (n=6) and validated by HPLC analysis.

Pharmacokinetic parameters for fluorocoxib D were evaluated following i.v. administration of 1  mg/kg over 20 min. Peak concentration of fluorocoxib D (114.8±50.5  ng/ml) was detected in plasma collected at 0.5 h after administration using a HPLC analysis as shown in Fig. 2. No fluorocoxib D was detected in plasma after 4 h following administration. The calculated pharmacokinetic parameters were shown as mean ± standard deviations (S.D.) in Table 5.

**Table 5:** Pharmacokinetic parameters (mean ± S.D.) of fluorocoxib D determined after i.v. 1  mg/kg administration in six research dogs.

Pharmacokinetic parameters	$C_{\max}$ (mg/L)	$t_{1/2}$ (h)	${\mathrm{AUC}}_{0\text{-}\infty }$ (mg*h/L)	${\mathrm{AUC}}_{0\text{-}8}$ (mg*h/L)	MRT (h)	Cl (L/kg*h)	${\mathrm{Vd}}_{\text{area}}$ (L/kg)
Fluorocoxib D (1 mg/kg, i.v.)	0.11±0.05	0.91±0.29	0.15±0.05	0.11±0.04	1.46±0.42	7.4±2.34	9.97±4.76

$C_{\max}$, maximum plasma concentration; $t_{1/2}$, terminal half-life; ${\mathrm{AUC}}_{0\text{-}\infty }$, area under the plasma concentration time curve from time 0 to infinity; ${\mathrm{AUC}}_{0\text{-}8}$, area under the plasma concentration time curve from time 0 to 8 h; MRT, mean residence time; Cl, clearance; ${\mathrm{Vd}}_{\text{area}}$, volume of distribution.

### 3.3 Specific Uptake of Fluorocoxib D in COX-2-Expressing 5637 Cells *In Vitro*

**Fig. 3:**
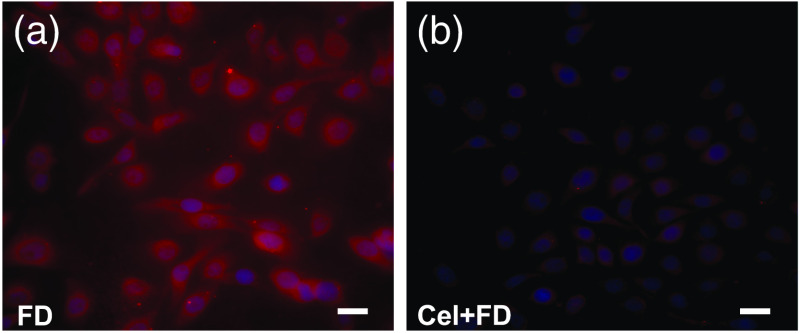
Specific uptake of fluorocoxib D by human bladder cancer 5637 cells *in vitro*. (a) 5637 cells were treated with 50 nM fluorocoxib D for 30 min. (b) 5637 cells were pretreated with 10  μM celecoxib for 30 min followed by 50 nM fluorocoxib D treatment for 30 min. After treatment, cells were washed three times with PBS, incubated for additional 1 h with fresh complete media, fixed and counterstained with DAPI (blue). 40× objective, scale bar 25  μm. FD, fluorocoxib D; Cel, Celecoxib.

To validate the specificity of fluorocoxib D uptake by human 5637 cancer cells, cells were pretreated with or without 10  μM celecoxib, a COX-2 selective inhibitor for 30 min, followed by 50-nM fluorocoxib D treatment for additional 30 min. Selective uptake of fluorocoxib D (red color) was detected in COX-2-positive 5637 cancer cells as shown in Fig. 3(a). Celecoxib prevented binding of fluorocoxib D to COX-2 enzyme in 5637 cancer cells as shown in Fig. 3(b). Nuclei of 5637 cells were counterstained with DAPI (blue) staining.

### 3.4 Specific Uptake of Fluorocoxib D in COX-2-Expressing Cancer Cells *In Vivo* in a Dog During Rhinoscopy-Guided Biopsy Procedure

**Fig. 4:**
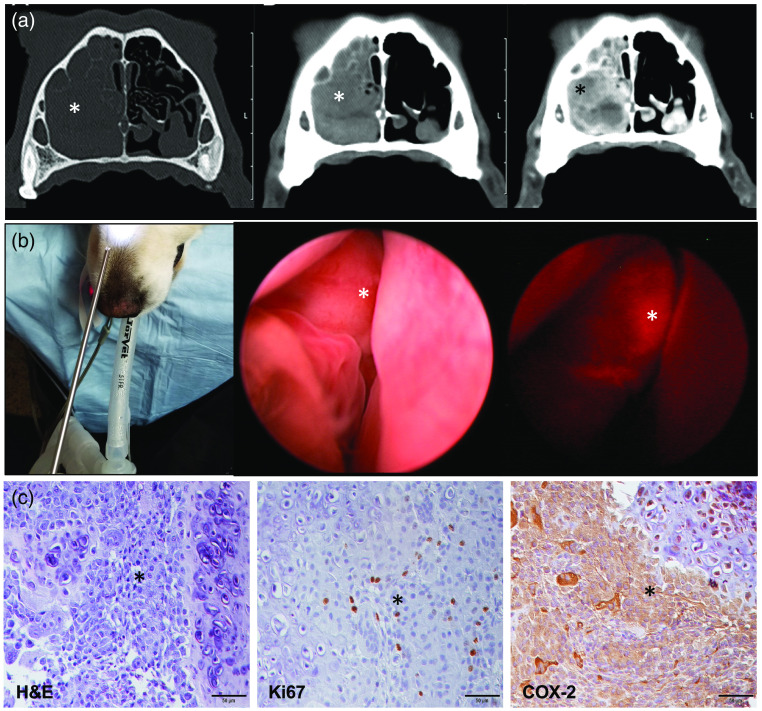
Fluorocoxib D uptake by canine HNC tumor during rhinoscopy *in vivo*. (a) Transverse CT images of the nose in a 9-year-old Golden Retriever displayed in a bone window (left panel; window center 600 HU, window width 2600 HU) and in a soft tissue window (window center 50 HU, window width 350 HU) before (middle panel) and after (right panel) intravenous contrast medium administration. HNC mass (asterisk) is associated with and fills nearly the entire right nasal cavity. There is extensive lysis of nasal turbinates and thinning of the nasal septum. On precontrast images, the mass is soft tissue attenuating and mildly heterogeneous (left and middle panels). Following contrast medium administration, there is heterogeneous contrast enhancement with multiple round hypoattenuating regions with rim enhancement, suggesting hemorrhage or necrosis (right panel). (b) Planning of insertion of rigid scope to nasal cavity before rhinoscopy procedure (left panel). A representative image of localized HNC mass (asterisk) by a white light (middle panel) and under fluorescence light (right panel) with specific uptake of fluorocoxib D (red color). (c) A representative histology images of H&E (left panel), Ki67 (middle panel), and COX-2 (right panel) protein expressions in HNC of chondrosarcoma cells (asterisk) by immunohistochemistry staining (brown color). 20× objective, scale bar 50  μm.

**Fig. 5:**
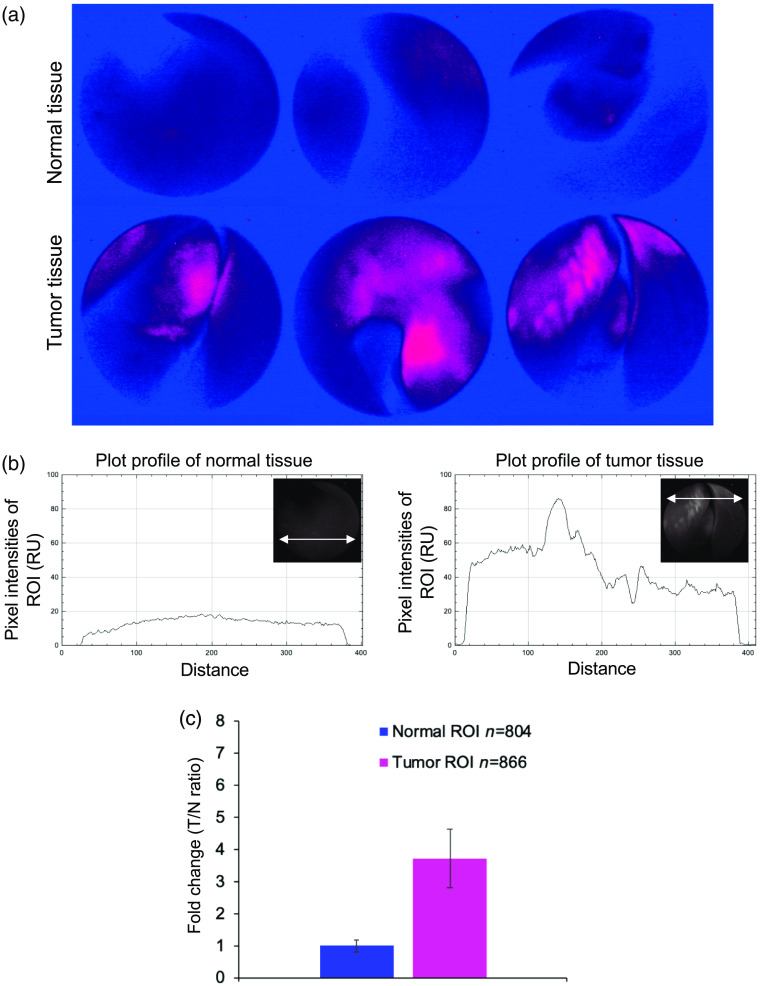
T/N ratio of fluorocoxib D by canine HNC tumor. (a) Representative heat map images of fluorocoxib D signal (pink color) from normal tissue (upper three images) and HNC tumor (lower three images) obtained during rhinoscopy using Adobe Photoshop CC2019 software (Adobe Acrobat). (b) Representative plot profile images of normal (left panel) and HNC tumor tissues using ImageJ software (NIH). (c) T/N ratio of fluorocoxib D signal obtained from normal (n=804) and tumor ROI (n=866) from plot profile images obtained from ImageJ software.

To validate the specific uptake of fluorocoxib D by COX-2-expressing cancer cells, we used a dog with naturally occurring and CT-identified HNC mass as a model of human cancer. In a dog CT revealed an ill-defined, lobular, mostly soft tissue attenuating, and mildly heterogeneous mass occupying almost the entire mid to caudal right nasal passage and resulting in extensive turbinate destruction as shown in Fig. 4(a). There was also evidence of early lysis and multifocal thinning of the nasal septum. The mass extended from the level of the right maxillary first premolar tooth to the level of the right fourth maxillary premolar tooth and filled the entire right maxillary recess. The mass displayed moderate heterogeneous contrast enhancement, with multiple rounded hypoattenuating regions with rim enhancement. The mass was visualized during rhinoscopy and directed biopsies were collected [Fig. 4(b)]. Specific uptake of fluorocoxib D was detected in HNC during rhinoscopy procedure in a dog as shown in Fig. 4(b) using white (middle panel) and fluorescence light exposures (right panel, red color). Histopathology analysis using H&E staining confirmed presence of HNC mass as a canine chondrosarcoma [Fig. 4(c), left panel]. The expression of COX-2 protein was confirmed in obtained HNC biopsy samples as shown in Fig. 4(c) (right panel, brown staining). The presence of Ki67-positive cells of HNC supported the evidence of proliferating cancer cells [Fig. 4(c), middle panel, brown staining]. T/N ratio of fluorocoxib D by canine HNC tumor was validated by ImageJ and Adobe PhotoShop CC2019 software as shown in Fig. 5. The representative heat map images of fluorocoxib D signal (pink color) from normal tissue (upper three images) and HNC tumor (lower three images) obtained during rhinoscopy using Adobe Photoshop CC2019 software (Adobe Acrobat) were shown in Fig. 5(a). The representative plot profile images of normal (left panel) and HNC tumor tissues using ImageJ software (NIH) were shown in Fig. 5(b). The averaged T/N ratio of fluorocoxib D signal obtained from normal (n=804) and tumor ROI (n=866) from plot profile images was in an average 3.7±0.9 as was shown in Fig. 5(c).

## 4 Discussion

The potential use of NSAID compounds as imaging agents has come under intense investigation.^25^^–^^30^ As previously published, several COX-2-targeted optical, SPECT, and PET imaging agents have been synthesized and shown to detect COX-2 expression in culture human tumor cell lines and/or in tumor xenografts in nude mice.^26^^,^^29^ Tested concentrations of fluorocoxib D of 1  mg/kg confirmed a safe administration with no clinically relevant adverse events based on physical examination, as well as whole blood, serum, and urine analysis. This was similar finding as we had with fluorocoxib A.^38^

Once NSAIDs are absorbed in blood stream, they are highly plasma-protein bound (80% to 97%) and have a relatively long elimination half-life.^48^^–^^51^ Indomethacin (Indocin) bioavailability is 100% when administrated orally, and ∼99% to 97% of indomethacin is bound to protein in plasma with an approximate $t_{1/2}$ of 0.3 to 4.5 h in various species.^52^ The elimination $t_{1/2}$ of fluorocoxib A was 11.0±2.5  h after i.v. administration of 1  mg/kg in healthy research dogs.^38^ This indicates higher stability of fluorocoxib A than indomethacin or robenacoxib (1.1 h after oral administration of 2  mg/kg).^53^ In contrast to fluorocoxib A, elimination $t_{1/2}$ of fluorocoxib D was 0.91±0.29  h after i.v. administration of 1  mg/kg in dogs. As compared to fluorocoxib A, which has a volume of distribution 18.3±3.7  ml/kg and is fast cleared from blood with a clearance of 0.94±0.64  L/kg*h after i.v. administration of 1  mg/kg,^38^ fluorocoxib D has even smaller volume of distribution 9.97±4.76  ml/kg and is cleared faster from blood with a clearance of 7.4±2.34  L/kg*h as shown in Table 5. No detectable levels of fluorocoxib D were found in any of the tested plasma samples after 4 h, suggesting that fluorocoxib D was degraded and eliminated from the blood at that time point.

HNC are among the most commonly diagnosed cancers in the United States and among the leading causes of cancer death.^54^^,^^55^ Early detection and more precise resection during surgery and biopsy procedures are key factors to improve patient’s survival rates. Spontaneous cancers in companion dogs offer a unique model for validation of imaging agents and devices for detection of human cancer. The histologic and biologic characteristics of many cancers in dogs are similar to those in humans.^38^^,^^40^^–^^46^ To this end, as a proof-of principal experiment, we evaluated fluorocoxib D, which specifically targets cancer cells using a dog with naturally occurring HNC. As seen in humans, COX-2-positive tumors in dogs^43^^,^^46^^,^^56^^–^^58^ show strong expression of the protein in the perinuclear area of the cells and in macrophages surrounding the tumors. Specific uptake of fluorocoxib D by COX-2-expressing cells was also confirmed *in vitro* using human 5637 cancer cells pretreated with celecoxib, the COX-2 selective inhibitor (Fig. 3). Fluorocoxib D specifically bound to COX-2-expressing canine HNC cells and allowed better visualization and identification of the COX-2-positive cancers as shown in Fig. 4(b) (right panel, red color). COX-2 expression was confirmed by IHC analysis of biopsy samples as shown in Fig. 4(c) (right panel, brown staining). Specific T/N ratio of detected fluorocoxib D signal was in an average of 3.7±0.9 using Image J analysis as shown in Fig. 5.

In conclusion, the results reported here demonstrate for the first time the safety and pharmacokinetic parameters of fluorocoxib D in healthy research dogs. In addition, fluorocoxib D uptake by HNC tumor with an average 3.7±0.9 T/N ratio signal *in vivo* was confirmed by immunohistochemistry staining for COX-2 expression in obtained HNC tumor biopsy samples. Our data suggest that fluorocoxib D might be a suitable candidate as the optical imaging agent for detection of COX-2-expressing HNC cancer to further assist with an image-guided minimally invasive biopsy procedure.
